# Reprogramming Fibrosis: How Protein PTMs Reshape the IPF Proteome

**DOI:** 10.3390/genes16111392

**Published:** 2025-11-20

**Authors:** Yunze Li, Wei Kong, Hanqi Zhang, Xinfeng Wei, Junxuan Yi, Mingwei Wang, Shunzi Jin, Duo Yu

**Affiliations:** 1 NHC Key Laboratory of Radiobiology, School of Public Health, Jilin University, Changchun 130012, China; liyz2722@mails.jlu.edu.cn (Y.L.); kongwei2722@mails.jlu.edu.cn (W.K.); 17862814170@163.com (X.W.); yijunxuan1993@163.com (J.Y.); wangmw23@mails.jlu.edu.cn (M.W.); jinsz@jlu.edu.cn (S.J.); 2 Department of Radiotherapy, China-Japan Union Hospital of Jilin University, Changchun 130033, China; zhanghq9922@mails.jlu.edu.cn

**Keywords:** post-translational modifications, idiopathic pulmonary fibrosis, lung injury, macro-phages, therapeutic strategies

## Abstract

Idiopathic pulmonary fibrosis (IPF) is a fatal and progressive lung disorder. Its pathological process involves persistent epithelial damage, ongoing inflammation, and dysregulated tissue repair. Currently, there are no effective treatment methods to improve patient survival. However, post-translational modifications (PTMs) have gradually garnered widespread attention. They are the processes by which various chemical groups are added to or removed from proteins’ amino acid side chains or the N- or C-terminal ends of the polypeptide chain following synthesis. Additionally, they can regulate the energy supply of cells, regulate the cell cycle, and affect important signaling pathways such as TGF-β. This review systematically summarizes different categories of PTMs, organizes the PTMs involved in various injury stages of IPF, outlines the roles of different cells throughout the process, and analyzes future clinical diagnosis and treatment strategies as well as intervention targets for IPF, providing guiding significance for the systematic intervention of IPF in the future.

## 1. Introduction

Idiopathic pulmonary fibrosis (IPF) is a progressive lung disease of unknown etiology with high incidence, high mortality, and substantial healthcare burdens worldwide [1]. It is worth noting that a diverse group of individuals, including those with asthma, allergic airway inflammation, pulmonary hypertension, and lung cancer patients who have undergone surgery or radiotherapy, are at high risk of pulmonary fibrosis. In contrast, diagnosing this condition is quite challenging as it necessitates the exclusion of known causes such as viral infections, autoimmune disorders, and genetic factors. Therefore, to frame the mechanistic background for PTM-targeted intervention in IPF, we summarize the common pathological features shared with other forms of Progressive Pulmonary Fibrosis (PPF), such as abnormal proliferation of fibroblasts, excessive deposition of the extracellular matrix (ECM), inflammatory injury, and tissue remodeling [2] while recognizing that a definitive diagnosis still requires systematic exclusion of known causes. Specifically, its progression can be summarized as three interrelated phases, which include epithelial injury, the inflammatory response, and fibrotic remodeling [3]. To start, the injury phase involves recurrent lung damage due to toxins, radiation, or drug toxicity. Impaired re-epithelialization by damaged Type II alveolar epithelial cells (AT2) initiates cascading responses, driving progression to inflammation [4]. Subsequently, during the inflammatory phase, inflammatory macrophages recruit neutrophils via cytokine release, which worsens alveolar damage [5,6]. The concurrent activation of nuclear transcription factors and the MAPK pathway increases the expression of profibrotic cytokines, particularly TGF-β. Paracrine signaling by these mediators amplifies macrophage recruitment and myofibroblast differentiation, which leads to the transition to the fibrotic phase [7]. Finally, in the stage of fibrotic remodeling, persistent fibroblast activation promotes the accumulation of the ECM. Moreover, uncontrolled collagen deposition and inflammation cause irreversible scar formation. Consequently, a definitive IPF diagnosis requires a multidisciplinary consensus that systematically rules out other interstitial lung diseases. Also, existing pharmacotherapies—pirfenidone and nintedanib—modestly decelerate functional decline but fail to arrest or reverse fibrosis [8,9], underscoring the urgency for mechanism-based interventions.

PTMs can greatly diversify protein functions and regulate biological processes, including cellular signaling, protein stability, and gene expression [10]. Indeed, more than 670 distinct PTMs have been catalogued to date, with phosphorylation, ubiquitination, glycosylation, and methylation among the most prevalent [11]. When it comes to the functions of these PTMs, exemplified by phosphorylation of glycogen phosphorylase [12] and acetylation of p53 [13], they can modulate enzymatic activity and transcription-factor function. Furthermore, as central regulators of cellular signaling, PTMs also affect protein fate and then coordinate physiological responses through signal integration and amplification [14]. For instance, ubiquitination can mark proteins for degradation [15], while glycosylation enhances protein stability [16] and facilitates signaling cascades [17] and chromatin remodeling [18]. It is particularly noteworthy that PTMs also play a role in IPF pathogenesis through fibroblast activation [19], ECM remodeling [20], and inflammatory modulation [21]. This review systematically evaluated how major PTMs influence the progression of IPF. We also incorporated newly identified PTMs, including lactylation, palmitoylation, and citrullination, to describe their stage-specific cellular effects and clinical potential, providing a mechanistic framework for PTM-targeted therapy for IPF.

## 2. Methods

### 2.1. Literature Search Strategy

A comprehensive literature search was conducted using the online databases PubMed (National Library of Medicine) and Web of Science. Published articles reported on the effects of PTMs on various cells during the stages of pulmonary fibrosis, including damage, inflammation, and repair, as well as their diagnostic and therapeutic applications in this condition. The keywords used included the individual use or a combination of these Medical Subject Headings (MeSH) terms: “idiopathic pulmonary fibrosis”, “post-translational modification”, “phosphorylation”, “ubiquitination”, “acetylation”, “methylation”, “glycosylation”, and so on. Boolean operators “OR” and “AND” were used to separate the synonyms and to string together the MeSH terms. Searches were restricted to research articles only, which could be accessed in full and written in English up to June 2025.

### 2.2. Screening and Selection of Studies

The initial screening of the retrieved studies was performed by Yunze Li to select studies with the following inclusion criteria: (1) reported PTMs in human IPF tissue, cells, or secretome and (2) used bleomycin-, silica-, or radiation-induced murine models explicitly presented as IPF surrogates and (3) conducted in-vitro experiments with primary human or mouse AT2 cells, fibroblasts, or macrophages under IPF-relevant stimuli (TGF-β1, ER stress, senescence) and (4) were published in English. The exclusion criteria were letters, editorials, notes, incomplete abstracts without full texts, those irrelevant to the scope, conference abstracts, duplicated studies, and irrelevant topics. All authors independently verified the screening of articles and checked the extracted data to avoid bias in selecting papers; this was performed between March and June 2025.

## 3. Classification and Functions of PTMs

PTMs, the crucial elements in cellular regulation, have significantly enriched the diversity of proteomic research. Common PTMs include phosphorylation, acetylation, and methylation. Even more, recent studies have revealed novel PTMs, including crotonylation, 2-hydroxyisobutyrylation, lactylation, dopamylation, and glutathionylation [22]. Here, we detailed the functional importance of these PTMs and their implications in lung pathology. 

### 3.1. Phosphorylation

Phosphorylation, originally reported in 1906 [23], remains the most intensively studied post-translational modification. By covalently tagging predominantly serine, threonine, and tyrosine residues [24], this reversible switch is thought to govern the functional output of roughly one-third of all cellular proteins [24,25,26]. Accordingly, it dynamically modulates protein activity and coordinates diverse cellular events. To illustrate, AKT1-mediated phosphorylation of malic enzyme 2 (ME2) at Ser9 promotes glycolytic enzyme complex formation, increasing metabolic flux [27]. Furthermore, CDK8/19 directly phosphorylates STAT5 Ser726, which triggers ILC2 secretion and elevates IL-5 and IL-13 cytokine levels. This cytokine-mediated regulation activates macrophages and fibroblasts, ultimately leading to collagen deposition [28]. Additionally, ROCK1/2 promotes fibroblast contraction and ECM synthesis by increasing MLC phosphorylation and Smad2/3 phosphorylation [29]. However, the highly selective ROCK2 inhibitor GNS-3595 reduces Smad2/3 C-terminal phosphorylation and then inhibits the fibroblast-myofibroblast transformation, which is induced by TGF-β1 [30]. Thus, phosphorylation orchestrates cellular energetics by modulating metabolic enzymes and signaling cascades. The phosphorylation of key proteins, such as Smad2/3 and STAT5, serves as the core switch that amplifies multiple signaling pathways, including TGF-β and ROCK. Targeting specific kinases or phosphorylation sites can influence processes such as ECM deposition and fibroblast-to-myofibroblast transformation.

### 3.2. Acetylation

Acetylation has two predominant forms, including N-terminal acetylation and reversible lysine acetylation regulated by KATs/KDACs [31,32]. In the context of the cell cycle and regulation of energy metabolism, elevated Ac-CoA levels stimulate Signal transducer and activator of transcription 3 (STAT3) and histone H4 acetylation, creating a feedforward loop that drives the proliferation of hepatocytes and liver regeneration, suggesting that acetylation can regulate the cell cycle [33]. The mitochondrial deacetylase SIRT3 modulates metabolic function, demonstrating the role of acetylation in energy regulation [34]. It should be noted that tubulin acetylation underpins the efficiency and directionality of intracellular transport, which is required to regulate cell survival [35]. Additionally, tubulin acetylation has been shown to stimulate the formation and transport of autophagosomes during starvation-induced autophagy [36,37]. Collectively, acetylation couples energy flux to autophagy induction and cell-cycle, thereby steering apoptosis, differentiation, and self-renewal.

### 3.3. Methylation

**Figure 1:**
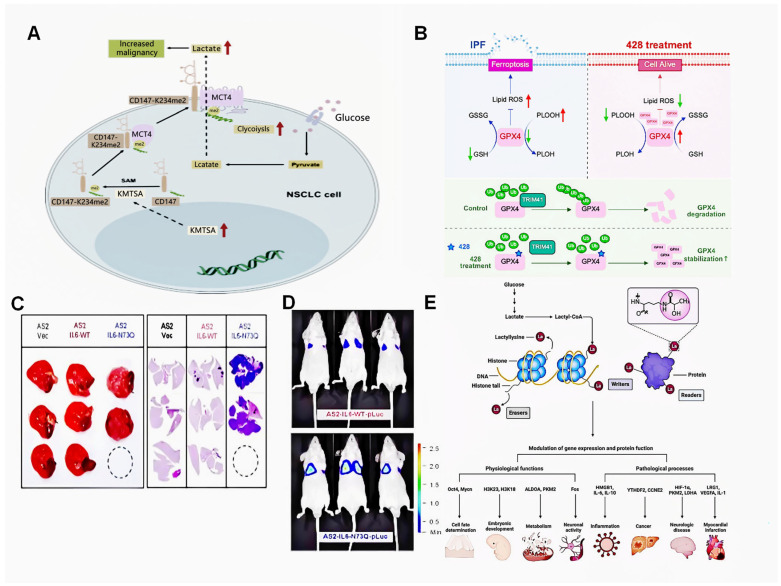
The impact of major PTMs such as methylation, ubiquitination, glycosylation, and lactylation on pulmonary diseases and their primary physiological functions. (**A**) Mechanism diagram of CD147 protein methylation modification accelerating the progression of non-small cell lung cancer (NSCLC). The red thick arrows represent an increase. (**B**) Mechanism diagram of the mechanism by which E3 ligase TRIM41 catalyzes GPX4 degradation, leading to uncontrolled ferroptosis and ultimately IPF. The red thick arrows represent an increase, the green thick arrrows represent a decrease. (**C**) Nude mice were injected via the tail vein with different cells. Gross anatomical inspection (left) and H&E-stained sections imaged with TissueFAXS (right) revealed necrotic lung regions. (**D**) In vivo metastasis of two different kinds of cells in nude mice. (**E**) Schematic illustration demonstrating how lactylation regulates gene expression and protein function, thereby influencing physiological and pathological processes. Figure (**A**) reprinted from Wang, Ke et al. (2021) [43]. Figure (**B**) reprinted from Zhang, Youping et al. (2025). [49] Figure (**C**,**D**) reprinted from Hung, Chun-Hua et al. (2024) Springer Nature [50]. Figure (**E**) reprinted from Hung, Hu, Yue et al. (2024) [51].

Methylation is catalyzed by methyltransferases, such as PRMT5/6 and METTL18/9, which transfer methyl groups from S-adenosylmethionine to target residues [38,39]. While lysine and arginine are primary targets [40], histidine and asparagine residues also undergo methylation [41,42]. In addition, lysine methylation can generate an acidic microenvironment that facilitates tumor proliferation (Figure 1A) [43]. Additionally, methylation regulates the cell cycle of cells [44]. Under hypoxic conditions, G9a methylates RUNX3 to activate genes associated with the cell cycle [45]. Beyond that, CBX5-recruited G9a deposits H3K9me2 at the RUNX3 promoter in primary IPF fibroblasts, silencing this cell-cycle brake and thereby sustaining fibroblast-to-myofibroblast conversion [46]. Moreover, in IPF, arginine methyltransferase PRMT5 and its methylated mark H3R2me2 are enriched at the FOXO1 promoter. Loss of PRMT5 function reduces FOXO1 expression and impairs the self-renewal of intrinsic fibrotic mesenchymal progenitor cells (MPCs), thereby exacerbating IPF [47]. In a few words, methylation maintains alveolar progenitor homeostasis and coordinates injury repair [48]. Such regulation prevents malignant transformation while promoting tissue regeneration by affecting the cell microenvironment and the cell cycle.

### 3.4. Ubiquitination

Ubiquitination is a dynamic PTM involving the sequential action of E1-E2-E3 enzymes, with E3 ligases specifically mediating isopeptide bond formation between the C-terminus of ubiquitin and the lysine residues of substrates [52]. It regulates protein localization, metabolic fate, and proteasomal degradation [53]. In IPF, TRIM41 acts as an E3 ligase to catalyze the K48 ubiquitin chain on GPX4, triggering its proteasomal degradation. This process leads to uncontrolled ferroptosis and subsequent collagen deposition (Figure 1B) [49]. Additionally, E3 ligase LMO7 is upregulated in IPF lung tissue, mediating K48 ubiquitination and degradation of SMAD7 at lysine 70. Thus, alleviating inhibition of TGF-βR1 and thereby enhancing TGF-β/SMAD signaling further promotes Epithelial–Mesenchymal Transition (EMT) [54]. To reiterate, biochemical screening revealed 53 genes that are dependent on ubiquitin, among which the core component of the APC/C complex, CDC20, and the HECT domain-containing protein ITCH, exhibit elevated expression. These proteins degrade E-cadherin and SMAD7 by ubiquitinating them, respectively, thereby jointly promoting EMT [55]. In summary, various E3 ubiquitin ligases target the core ferroptosis proteins GPX4 and the TGF-β signaling modulators SMAD7 by attaching K48-linked ubiquitin chains. This disruption of protein homeostasis triggers either collagen deposition or EMT.

### 3.5. Glycosylation

Glycosylation, with the main types being N-glycosylation and O-glycosylation [56,57]. Specifically speaking, N-glycosylation modifies asparagine residues, while O-glycosylation attaches sugars to serine or threonine [58]. Studies have found that TGF-β-driven PSAP hyperglycosylation disrupts chaperone binding and lysosomal targeting [59]. Similarly, Fucosyltransferase 4 (4FUT) increases fucosylation modifications on L1 cell adhesion molecule (L1CAM), which leads to abnormalities in the AR-*FUT4*-L1CAM-AJ signaling pathway [60]. Furthermore, properly glycosylated MUC6 mediates cell-matrix interactions through glycan–clusterin binding, maintaining the integrity of the gastric mucosa [61]. In terms of lung pathology, long-term treatment with EGFR-TKI increases deglycosylated IL-6 (Figure 1C,D). Following this, there was a significant increase in metastatic lesions and the enhancement of the metastatic potential of AS2-IL6-N73Q-pLuc cells. This indirectly leads to pulmonary fibrosis [50]. To sum up, glycosylation can change the folding of proteins and affect cell adhesion and signaling pathways, thus affecting cell metastasis and interaction.

### 3.6. SUMOylation

SUMOylation involves three major paralogs (SUMO1–3) that modify target proteins post-translationally [62,63]. The dynamic process requires the coordinated action of E1 (SAE1/2), E2 (UBC9), and E3 enzymes for substrate-specific modification [64]. SUMOylation can influence inflammation and regulate the course of biological activities. In detail, SUMOylation at lysines 113, 161, and 257 of Annexin-A1 accelerates autophagic clearance of IKKα, thereby dampening pro-inflammatory mediator release in microglia subjected to OGD/R insult [65]. Also, it suppress Type I Interferon (IFN-1) responses, which can be therapeutically reversed by TAK-981 [66]. Likewise, in NSCLC, SUMOylated hnRNPA2B1 activates ALIX via SIM interaction, driving extracellular vesicle-mediated lymphangiogenesis and metastasis [67]. In short, SUMOylation primarily functions in the regulation of inflammatory factor release and lymphatic angiogenesis during inflammatory responses.

### 3.7. Lactylation

Lactylation is a novel PTM that is induced by lactic acid accumulation during metabolism and occurs mainly at lysine residues of histones, such as H3K18 and H3K27. L-lactylation is involved in the regulation of several life activities (Figure 1E), including uterine remodeling [68] and neuronal stress [69,70]. It’s worth mentioning that Li et al. reported that Glis1-mediated glycolytic flux increases lactate and acetyl-CoA levels, increasing H3K18la/H3K27Ac at pluripotency genes to facilitate cellular reprogramming [71]. Furthermore, lactylation drives the proliferation of smooth muscle cells via HIF-1α target gene regulation and metabolic reprogramming in pulmonary hypertension [51]. Undoubtedly, the role of lactylation in metabolic reprogramming is not negligible in many systems, such as the nervous, reproductive, and respiratory systems.

### 3.8. Other PTMs

Beyond these principal PTMs, additional modifications occur—such as palmitoylation, which covalently links palmitate to cysteine residues through thioester bonds [72,73]. It integrates metabolism, such as glycolytic flux [74], with cellular energetics [75,76]. Ultimately, it promotes the transfer of the NLRP3 inflammasome and pyroptotic signaling [73,77]. Moreover, succinylation is the process by which a succinyl donor covalently binds a succinyl group to a lysine residue [78]. It serves as a critical hub for maintaining mitochondrial function [79] and cellular energy supply [80]. Recently, research on citrullination in waveform proteins (Vimentin) has advanced considerably. It converts arginine residues in proteins to citrulline [81]. During inflammation, citrullinated vimentin acquires neo-epitopes that breach immune tolerance, provoking autoreactive antibody production and sustained inflammatory cascades [82]. With the rapid development of science, crotonylation, glutathionylation, and so on have been discovered. Their role in pulmonary fibrosis is also being recognized (Table 1).

On the whole, numerous PTMs regulate fundamental cellular processes and may contribute to the three stages of pulmonary fibrosis. The reason can be ascribed to the following aspects, beginning with, in terms of cellular energy supply, phosphorylation, acetylation, and succinylations all influence mitochondrial function, the energy supply hub. Afterwards, regarding cellular metabolism and differentiation, these PTMs, including phosphorylation, acetylation, ubiquitination, and methylation, can regulate cell cycle progression. Beyond that, sumoylation, acetylation, and palmitoylation further modulate autophagy and pyroptosis pathways. These mechanisms are significant in aplastic regeneration and EMT in pulmonary fibrosis AT2 cells. Additionally, in inflammatory responses, methylation and lactylation shape the immune microenvironment, while sumoylation, palmitoylation, and citrullination facilitate inflammatory factor secretion through inflammation bodies, thereby promoting inflammatory cascades. Finally, ubiquitination, palmitoylation, and glycosylation regulate the homeostasis of biomolecules like proteins and lipids, as well as intercellular interactions. We hypothesize that PTMs are indispensable in the remodeling of the ECM and the development of fibrosis. Next, we discuss the specific effects of PTMs on IPF in more detail, considering recent research findings and the examination of different cells at various stages of the disease.

## 4. Effects of PTMs on Various Stages of IPF

Multiple cells are involved in the pathological process of the three stages of pulmonary fibrosis. Primarily, in injury, alveolar epithelial cells (AECs), especially AT2 cells, cannot complete normal re-epithelialization [83]. Afterwards, during the inflammatory response stage, macrophages, neutrophils, and lymphocytes release a range of pro-inflammatory cytokines, which in turn recruit fibroblasts to the site of injury. Finally, during the repair stage, epithelial cells undergo EMT, acquire mesenchymal properties, and transform into cells with a fibroblast phenotype. Beyond that, fibroblasts are activated by cytokines, growth factors, and mechanical stress to differentiate into myofibroblasts. In contrast to fibroblasts, myofibroblasts have a greater ability to synthesize ECM, and their continued activation and proliferation lead to the continued progression of pulmonary fibrosis [84]. Without reservation, PTMs are crucial in regulating cellular functions involved in all three phases of lung fibrosis: injury, inflammation, and repair.

### 4.1. Injury Stage

**Figure 2:**
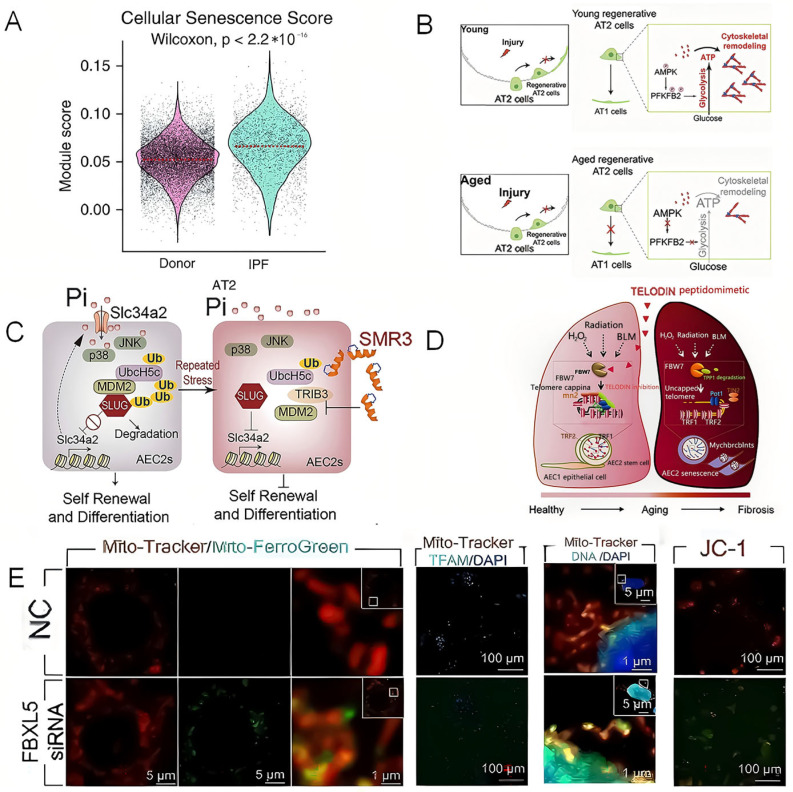
Phosphorylation and ubiquitination, among other PTMs, impact the self-renewal and activity of AT2 cells, thereby influencing pulmonary fibrosis. (**A**) Violin plot visualization of the cellular senescence score, calculated from core senescence genes in the AT2-cell subset of human epithelial single-cell RNA-seq data. (**B**) Schematic illustration affecting the conversion of AT2 to AT1. The process of cellular transformation and metabolic changes in young and aged lungs following injury, with a focus on the role of AMP-activated protein kinase (AMPK) and 6-phosphofructo-2-kinase/fructose-2,6-bisphosphatase 2 (PFKFB2) in regulating glycolysis and cytoskeletal remodeling. (**C**) SLUG-mediated signaling pathway mechanism diagram affecting AT2 cell self-renewal. (**D**) Schematic diagram of the mechanism of FBW7 mediating aging and pulmonary fibrosis through amplifying the stem cell pool of mouse alveolar epithelial cells (AEC2). (**E**) Immunofluorescence staining and JC-1 assay show mitochondrial dysfunction after FBXKL5 knockdown in MLE-12 cells. Figure (**A**) reprinted from Yao, Changfu et al. (2021) [85]. Figure (**B**) reprinted from Wang, Zheng et al. (2023) [86]. Figure (**C**) reprinted from Lv, Xiaoxi et al. (2023) [87]. Figure (**D**) reprinted from Wang, Lihui et al. (2020) [88]. Figure (**E**) reprinted from Shao, Min et al. (2024), Ivyspring International Publisher [89].

Lung epithelial cells are the most crucial initiating cells of pulmonary fibrosis, which consist of two principal subtypes, including squamous Alveolar Type I Cells (AT1) and cuboidal AT2 cells. Among them, AT2 cells are the progenitor cells. They can be differentiated into AT1 cells, preventing pulmonary fibrosis [83]. Yet after lung injury, it shows that AT2 cells from IPF patients carry markedly higher senescence scores than donor cells (Figure 2A,B) [85,86]. Notably, PTMs can regulate the function of AEC2 in various ways, which in turn affects pulmonary fibrosis. Specifically speaking, PTMs can trigger pulmonary fibrosis by affecting the conversion of AT2 to AT1. The transcriptional repressor SLUG can accumulate in AT2 cells and inhibit their self-renewal and differentiation into AT1 cells. We found that the elevated SLUG represses the expression of the phosphate transporter SLC34A2 in AT2s, which reduces intracellular phosphate and represses the phosphorylation of JNK and P38 MAPK, two critical kinases supporting lung alveolar regeneration (LAR), leading to LAR failure (Figure 2B,C). Beyond that, TRIB3, a stress sensor, interacts with the E3 ligase MDM2 and inhibits the ubiquitylation degradation of SLUG, thereby exacerbating lung fibrosis [87]. Simultaneously, PTMs can also lead to cell repair deficiency by affecting the activity of AT2 cells. In my view, there are two common ways, including telomere uncapping and ferroptosis. More specifically, the E3 ubiquitin ligase FBW7 binds to telomere protection protein 1 (TPP1), which promotes TPP1 multisite polyubiquitination and accelerates its degradation, triggering telomere uncapping and the DNA damage response (Figure 2D) [88]. Additionally, after ubiquitin ligase FBXL5 knockdown, mitochondrial iron deposition was observed using confocal microscopy. Furthermore, TFAM protein levels decreased while mtDNA release increased, Δψm significantly declined, suggesting impaired mitochondrial function (Figure 2E). Finally, impaired mitochondrial function leads to increased intracellular oxidative stress, thereby promoting cellular damage [89]. Except during the injury, epithelial cells are also instrumental in the repair phase; they can be transformed into cells with a fibroblast phenotype by acquiring the properties of MSCs through EMT [90]. What’s more, PTMs affect lung fibrosis by influencing EMT. Specifically, SUMOylated SMAD4 contributes to TGF-β. To elaborate, SENP1 fine-tunes TGF-β signal transduction by catalyzing the de-SUMOylation of pivotal signaling molecules such as Smad4 and Ras, thereby modulating their activity and downstream responses. Additionally, the lung-enriched integrin α3β1 (ITA3) mutation A349S leads to increased glycosylation, which, in turn, interferes with the biosynthesis of ITA3. All two factors may promote EMT and drive lung fibrosis [91].

Restating the above, alterations in PTMs targeting epithelial cell proteins are the critical determinants of the injury stage. Various PTMs, including phosphorylation, ubiquitination, glycosylation, and SUMOylation, can affect lung fibrosis by targeting lung epithelial cells. During the injury, the impact of PTMs on epithelial cells is primarily manifested in ubiquitination, including the inhibition of ubiquitin-mediated degradation of the transcriptional repressor SLUG, thereby impeding the conversion of AT2 to AT1. Meanwhile, ubiquitination can also directly affect the activity of AT2 cells by influencing telomere uncapping through ubiquitin ligases and promoting ferroptosis, ultimately contributing to the progression of pulmonary fibrosis. Whereas during repair, phosphorylation, SUMOylation, and glycosylation drive EMT, thereby propagating fibrotic remodeling. Consequently, targeting lung epithelial cells and corresponding PTMs for treating pulmonary fibrosis has important potential therapeutic significance.

### 4.2. Inflammatory Response Stage

During the inflammatory phase, multiple cell types are involved in the initiation. These cells include macrophages, regulatory T cells (Treg), neutrophils, and monocyte-derived macrophages (MoDMs). They also recruit and activate other immune cells by secreting cytokines, chemokines, and inflammatory mediators, exacerbating the inflammatory response and tissue damage.

#### 4.2.1. Macrophages

Foremost, macrophages rapidly infiltrate the injury site, serving as first-responders in the inflammatory cascade. They can differentiate into pro-inflammatory M1-type macrophages and profibrotic M2-type macrophages [92,93,94]. PTMs promote pulmonary fibrosis by promoting macrophage phenotype transformation. To illustrate, chronic lung injury inactivates the ubiquitin editing enzyme A20, leading to the accumulation of the transcription factor C/EBPβ in alveolar macrophages, which in turn upregulates many profibrotic factors [95]. Subsequently, M1 macrophages shift toward a pro-fibrotic M2 phenotype. Then, as fibrosis evolves, macrophage oxidative stress releases abundant ROS and RNS [96], and also exacerbates the inflammatory response, promotes the synthesis of the ECM [97]. It is worth mentioning that phosphorylation targets the transport protein SLC15A3 in macrophages, which binds to the scaffold protein p62, thereby regulating the antioxidant axis composed of p62-NRF2, and directly controls the generation of ROS [98]. This suggests that PTMs can also affect pulmonary fibrosis by affecting macrophage oxidative stress. Besides macrophage phenotypic shifts and oxidative stress, macrophage pyroptosis affects pulmonary fibrosis. Macrophage pyroptosis is a form of programmed cell death characterized by cell expansion, cell membrane rupture, and the release of inflammatory factors [99]. For example, a cigarette smoke (CS)-induced increase in lactate may drive NLRP3 inflammatory vesicle-dependent macrophage pyroptosis by increasing histone lactylation levels [100]. As a result, the development of fibrosis in pulmonary fibrosis is influenced by the regulation of the synthesis and remodeling of the ECM [101]. All in all, macrophages are rapidly recruited to injured alveoli, where their phenotype, oxidative milieu, and mode of death are decisively shaped by PTMs. Ubiquitination of A20, phosphorylation of SLC15A3, and histone lactylation have been shown to drive macrophage phenotype transformation, amplify ROS/RNS generation, and trigger NLRP3-mediated pyroptosis, respectively. Each of these PTM-driven events escalates extracellular-matrix synthesis and, consequently, fibrotic progression. Collectively, the data indicate that targeting PTMs in macrophages represents a tractable anti-fibrotic strategy.

#### 4.2.2. Regulatory T Cells

Tregs play complex and multifaceted roles in the inflammatory phase of pulmonary fibrosis. Treg can secrete TGF-β, a key cytokine in pulmonary fibrosis, promoting the initiation and progression of fibrosis during the early inflammatory stage [102]. What is widely acknowledged is that FOXP3 serves as a key transcription factor for Treg cells, and its stability directly influences their function. Further, PTMs, such as ubiquitination, acetylation, and phosphorylation, can regulate the stability of FOXP3. Specifically speaking, ubiquitination may lead to the degradation of FOXP3, whereas hypoxia-inducible factor 1 (HIF1) can induce K48-linked polyubiquitination of FOXP3, triggering its proteasomal degradation [103]. In contrast, silencing the E3 ligase STUB1 stabilizes FOXP3 and boosts Treg suppressive function [104]. In contrast to ubiquitination, acetylation promotes the interaction of FOXP3 with chromatin, thereby enhancing its transcriptional regulatory function [105,106]. In addition, glycyrrhetinic acid may alleviate radiation-induced pulmonary fibrosis by inhibiting TGF-β1 secretion from Treg cells through the modulation of fibrosis-related PTMs [107]. In summary, the profibrotic potential of Treg is primarily exerted via the release of TGF-β and is regulated by the post-translational dynamics of FOXP3. Ubiquitination, notably K48-linked polyubiquitination mediated by HIF-1, destabilizes FOXP3, whereas acetylation and the depletion of STUB1 extend its half-life and chromatin interaction. Consequently, pharmacological modulation of these PTMs, such as the suppression of TGF-β mediated by glycyrrhetinic acid, is increasingly recognized as a viable strategy to inhibit fibrosis driven by Tregs.

#### 4.2.3. Other Immune Cells

In addition to macrophages that are first involved in the inflammatory phase and Treg that can secrete TGF-β, there are plenty of other immune cells involved. For instance, neutrophils act as potent effectors of tissue injury during pulmonary fibrosis by deploying neutrophil extracellular traps (NETs) at inflammatory foci [108]. Furthermore, the formation of NETs is closely associated with peptidylarginine deiminase 4 (PAD4)-mediated histone citrullination, a citrullinated modification that exacerbates tissue damage and recruits fibroblasts, thereby accelerating the progression of pulmonary fibrosis [109]. Similar to neutrophils, PTMs can lead to the accumulation of MoDMs in pulmonary fibrosis. They exert their functions at sites of inflammation and tissue injury by secreting cytokines and chemokines, thereby promoting fibroblast activation and ECM deposition [110]. As an illustration, histone monomethylation at RAP1A regulatory elements enhances RAP1A expression. This facilitates the sustained accumulation of MoDMs, subsequently inducing the expression of ACSL4, a ferroptosis-promoting gene, in lung epithelial cells. These events collectively drive the inflammatory phase of pulmonary fibrosis [111].

PTMs reprogram tissue-infiltrating leukocytes into profibrotic drivers. Macrophages rapidly enter injured alveoli, where A20 ubiquitination pushes them toward the M2 phenotype, SLC15A3 phosphorylation amplifies reactive oxygen species, and histone lactylation triggers NLRP3-mediated pyroptosis; each event escalates ECM deposition. Treg secrete TGF-β only when their master transcription factor FOXP3 remains intact; HIF-1-mediated ubiquitination shortens FOXP3 half-life, whereas acetylation or STUB1 depletion prolongs it, and agents such as glycyrrhetinic acid exploit this balance to suppress fibrosis. Neutrophils release PAD4-citrullinated extracellular traps that damage tissue and recruit fibroblasts, while MoDMs accumulate after RAP1A enhancer histone monomethylation and sustain inflammation by secreting cytokines and driving epithelial ferroptosis. Targeting these modification events, therefore, offers a multi-hit strategy against pulmonary fibrosis.

### 4.3. Repair Stage

During the repair phase, multiple cell types, including fibroblasts, lung-resident mesenchymal stem cells (LR-MSCs), and myofibroblasts, are involved in tissue repair and fibrosis. These cells promote the synthesis and deposition of ECM through complex interactions and signaling, leading to the structural remodeling and dysfunction of tissues.

#### 4.3.1. Fibroblasts

Chiefly during pulmonary fibrosis repair, TGF-β drives fibroblast conversion to myofibroblasts [112]. As mentioned earlier, it’s secreted by Treg. This signaling molecule activates downstream signaling pathways, mainly the Smad-dependent pathway and the non-Smad pathway, which are the central drivers of fibrotic remodeling [113,114,115]. Many PTMs can affect TGF-β, which in turn leads to fibroblast activation and excessive deposition of the ECM. Among them, phosphorylation, lactylation, SUMOylation, and ubiquitination can impact the Smad-dependent pathway and then affect TGF-β. In this regard, tartrate-resistant acid phosphatase 5 (ACP5) [116] and histone lactylation caused by PM2.5 [117] can upregulate TGF-β1 in a TGFβR1/Smad3-dependent manner. Moreover, elevated lactate fuels CBP/p300-catalyzed histone lactylation primarily H3K18la and H3K9la [118,119,120,121]. In alveolar epithelial cells, heightened H3K18la boosts TGF-β1 release via the YTHDF1/m6A/NREP axis [119]. Whereas in fibroblasts, increased H3K9la induces methyltransferase-like 3 (METTL3) transcription, enhancing METTL3-mediated m6A modification of lncNONMMUT062668.2 (lnc668) [121]. Together, the resulting TGF-β1 and m6A-lnc668 drive fibroblast-to-myofibroblast differentiation during pulmonary fibrosis [119,121]. Additionally, PIAS4-mediated enhancement of Smad3 SUMOylation [122] and DUOX1-mediated degradation of Smad3 ubiquitination [123] can also affect TGF-β. Also, Ginkgolic acid (GGA) can inhibit deacetylase activity [124,125]. H3K18la boosts YTH N6-methyladenosine RNA binding protein F1(Ythdf1) m^6^A-reader gene transcription [119]. Galactoglucan lectin-3 binds to the αv integrin, αvβ1, αvβ5, αvβ6, and TGFβRII subunits in a glycosylation-dependent manner [126,127]. All of the above factors can modulate TGF-β1-dependent fibroblast activation, inflammatory cell accumulation, and pro-inflammatory cytokine secretion. They also facilitate the transformation of fibroblasts into myofibroblasts. Moreover, the glycolytic enzyme PKM2 tetramer enhances TGF-β1 signaling through direct binding of Smad7 to its MH2 structural domain, which interferes with the interaction between Smad7 and the TGF-β type I receptor (TβR1), reduces TβR1 ubiquitination, stabilizes TβR1, and affects lung fibrosis [128]. Furthermore, Palmitoylation affects Cd-dependent fibrotic signaling by increasing cellular Cd accumulation and supporting the post-translational processing of TGFβ1-dependent proteins [129]. Overall, phosphorylation, lactylation, ubiquitination, acetylation, glycosylation, and palmitoylation converge on the TGF-β axis to control fibroblast fate. These modifications upregulate TGF-β1 ligand expression, enhance Smad3 activity, stabilize TβR1, and block Smad7-mediated receptor degradation, thereby accelerating myofibroblast conversion and ECM deposition. Nevertheless, SUMOylation inhibits TGF-β signaling and attenuates fibrosis. Consequently, TGF-β-directed PTMs constitute high-value, druggable nodes for antifibrotic therapy.

Another key point to remember is that fibroblasts do not promote pulmonary fibrosis only by transforming into myofibroblasts, their senescence can also promote pulmonary fibrosis [130,131]. Specifically speaking, V^+5^-dependent fibroblast senescence is associated with the increase of S-glutathionylation [132]. As demonstrated above, fibroblasts are derived from AT2 [133,134]. In addition, they interact in lung fibrosis. Cytokines such as TGF-β secreted by AT2 cells can activate fibroblasts, prompting them to transform into myofibroblasts [135]. Consequently, PTMs can exploit their crosstalk to intensify fibrosis. In AEC2, H3K18 lactylation up-regulates H3K18la-marked transcription, boosting TGF-β1 release and driving fibroblast-to-myofibroblast conversion. Elevated lactate further elevates global lactylation in fibroblasts, accelerating their myofibroblastic transition and worsening pulmonary fibrosis [119].

Restating the above, Phosphorylation, acetylation, glycosylation, ubiquitination, palmitoylation, SUMOylation, S-glutathionylation, and lactylation influence fibroblast senescence, transformation into myofibroblasts, and interactions with AT2 cells, thereby shaping the progression of pulmonary fibrosis. In these processes, PTMs significantly impact pulmonary fibrosis by modulating the activity and signaling pathways of TGF-β. Thus, TGF-β-directed PTMs represent tractable, high-value targets for antifibrotic therapy.

#### 4.3.2. LR-MSCs

Emerging evidence indicates that myofibroblasts arise from epithelial cells, fibroblasts, and even LR-MSCs, thereby hastening IPF progression [136,137]. SUMOylation can influence the transformation of LR-MSCs into myofibroblasts. For instance, the overexpression of SENP1 in LR-MSCs can stimulate the WNT/β-catenin and Hedgehog/glioma-associated oncogene homolog (GLI) signaling pathways through de-SUMOylation of key proteins and promote the transformation of LR-MSCs into myofibroblasts, exacerbating the development of lung fibrosis [138].

#### 4.3.3. Myofibroblasts

Myofibroblasts are activated fibroblasts with the characteristics of smooth muscle cells. They can synthesize and secrete large amounts of ECM and also remodel tissue structure through their contractile ability, leading to stiffness and dysfunction of lung tissue [139]. PTMs can also affect the number and function of myofibroblasts, which in turn affects lung fibrosis. As an illustration, acetylation of histone H3 lysine at position 27 (H3K27ac) can activate the opening of the chromosomal region in the LINC00941 promoter. The transcription factor ATF3 binds to this region, and LINC00941 transcription increases. Following this, highly upregulated LINC00941 accelerates lung fibrosis by promoting the proliferation and migration of myofibroblasts [140]. Conversely, TSA elevates acetylation of both histone and non-histone proteins by inhibiting histone deacetylase (HDAC) activity. TSA treatment inhibits the transcription and translation of smooth muscle α-actinin (α-SMA), a cytoskeletal protein predominantly secreted by myofibroblasts, which is a critical process in the pathogenesis of IPF [141,142]. To summarize, in the final stage of repair, PTMs can influence the synthesis and deposition of ECM, and the remodeling of tissue structure and function by affecting the conversion of EMT cells, fibroblasts, and LR-MSCs to myofibroblasts and consequently the synthesis and deposition of ECM. As a result, acetylation affects myofibroblasts during the repair stage, and H3K27ac can promote pulmonary fibrosis and consequently generate targeted therapeutic TSAs, which have satisfactory therapeutic efficacy and clinical potential for pulmonary fibrosis.

Collectively, PTMs have a wide range of effects on cellular functions at different stages of pulmonary fibrosis, including influencing the differentiation of epithelial cells during the injury stage. Modulating immune cells to influence the inflammatory response during the inflammatory stage. Regulating fibroblast activation, ECM remodeling, and fibrosis during the repair stage. By illuminating how PTMs drive the initiation and progression of pulmonary fibrosis, these findings establish a robust framework for identifying novel diagnostic biomarkers and therapeutic targets. Future studies may reveal the specific applications of PTMs in pulmonary fibrosis and assess their potential for clinical application (Table 2).

## 5. Diagnosis and Treatment of IPF

Authors should discuss the results and how they can be interpreted in light of previous studies and the working hypotheses. The findings and their implications should be addressed in the broadest context possible. Future research directions may also be highlighted.

As mentioned above, IPF is a complex chronic disease with a pathogenesis that involves multiple cellular and cytokine interactions. These include the activation of fibroblasts, the activation of numerous inflammatory pathways, and the excessive deposition of the ECM. As the disease progresses, severe pulmonary fibrosis not only impairs gas exchange but is also accompanied by life-threatening complications, including pulmonary hypertension, right heart failure, and respiratory failure. Therefore, early diagnosis and aggressive treatment are essential to slow disease progression and improve patient prognosis. However, completely reversing this fibrotic lesion is difficult once it has developed. Current therapeutic agents focus on slowing disease progression but are unable to cure it. Based on the above studies, we found that PTMs strongly affect the development of pulmonary fibrosis at various stages. Therefore, we reviewed the value of PTMs in the diagnosis and treatment of pulmonary fibrosis through the application of protein PTMs.

### 5.1. Diagnosis of IPF

IPF is often severe and irreversible once it occurs; early diagnosis is crucial. At this stage, the most common clinical diagnosis of pulmonary fibrosis is based on its typical symptoms combined with imaging. However, it is not accurate enough and lacks more precise diagnostic markers. Nowadays, PTMs are emerging as promising diagnostic indicators for pulmonary fibrosis. To a certain extent, IPF tissues can not only exhibit markedly reprogrammed protein abundance alongside shifted acetyl- and succinyl-proteome signatures [144], but also resolve the clinical challenge of differentiating between two diseases with similar symptoms. As is well known, separating IPF from connective-tissue-disease–associated ILD (CTD-ILD) remains challenging. However, emerging data indicate that advanced glycation end-products (AGEs) can serve as discriminatory biomarkers for this distinction [145]. Furthermore, many protein PTMs can help diagnose IPF, such as TPI1 dopaminylation [146], H3K18 lactylation [119], RAP1A monomethylation [111], Spry1 ubiquitination [147], and the related protein, ubiquitin aldehyde-binding protein Otubain1 (OTUB1) [148]. Obviously, PTMs are emerging as valuable tools for diagnosing pulmonary fibrosis. Altered levels of acetylation, succinylation, and dopaminylation are intimately linked to the pathological mechanisms that drive this disease. Additionally, altered glycosylation patterns alone can effectively discriminate IPF from CTD-ILD, offering a specific biomarker for differential diagnosis. In a word, as potential biomarkers of pulmonary fibrosis, PTMs increase the accuracy of clinical diagnosis. However, the application of PTMs in clinical diagnostics is relatively rare, and future studies should focus on bridging the gap between laboratory studies and clinical applications.

### 5.2. Treatment of IPF

Subsequently, patients diagnosed with pulmonary fibrosis require appropriate treatment. Treating pulmonary fibrosis is challenging. Current treatments include pharmacotherapy, oxygen therapy, and rehabilitation. Although these methods can improve the quality of life and symptoms of patients to a certain extent, their effects are relatively limited. For end-stage patients, lung transplantation may be the only cure, but it faces problems such as donor shortages and postoperative immune rejection. However, beyond their diagnostic value in pulmonary fibrosis, PTMs also inspire innovative treatment paradigms when integrated with clinical therapies and precision-targeted strategies.

**Figure 3:**
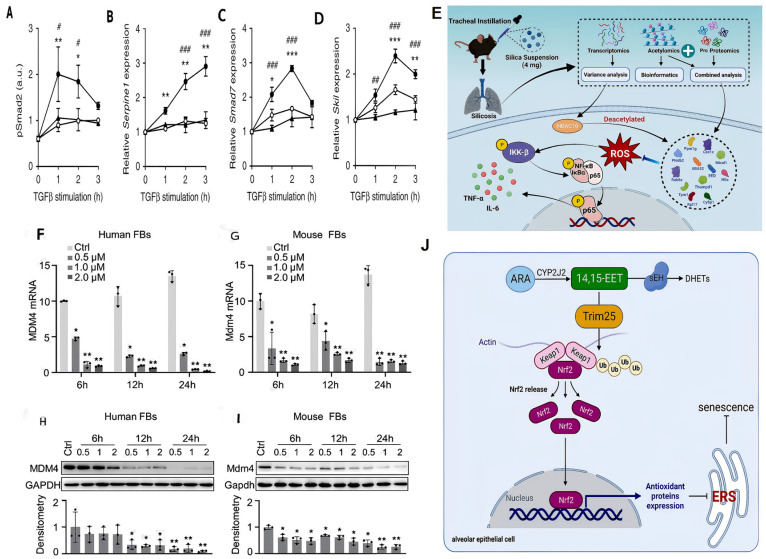
Schematic diagram of the role of PTMs in the pharmacological treatment of IPF, therapies targeting key signaling pathways of pulmonary fibrosis, therapies targeting key cells, and novel therapeutic approaches. (**A**–**D**) Alveolar type 2 cells were dissociated from doxycycline-induced conditional Nedd4-2^ȡ/ȡ^ mice and control mice. After pretreatment with 1 ng/mL TGF-β, the levels of phosphorylated Smad2 (pSmad2) and the transcriptional expression of TGF-β-regulated genes were measured. * *p* < 0.05, ** *p* < 0.01, *** *p* < 0.001 compared to control mice. # *p* < 0.05, ## *p* < 0.01, ### *p* < 0.001 compared to pirfenidone treated conditional Nedd4-2^−/−^ mice. (**E**) Schematic illustration showing that aerosolized LPAE-HDAC10 suppresses acetylation-mediated ROS/NF-κB signaling to treat pulmonary fibrosis. (**F**–**I**) Expression levels of Fas, p53, and acetylated p53 Ac-p53. *, *p* < 0.05; **, *p* < 0.01. (**J**) A mechanism diagram of EETs treating pulmonary fibrosis via the Trim25/Keap1/Nrf2 axis. Figure (**A**–**D**) reprinted from Duerr, Julia et al. (2020) Springer Nature [149]. Figure (**E**) reprinted from Tian, Yunze et al. (2023) [153]. Figure (**F**–**I**) reprinted from Mei, Qianru et al. (2023), Ivyspring International Publisher [154]. Figure (**J**) reprinted from Zhang, Chen-Yu et al. (2023) [155].

When it comes to clinical treatments, the most commonly used are pharmacological treatments, whose effects are focused mainly on slowing disease progression. In particular, PTMs are indispensable in the mechanism of action of pirfenidone and nintedanib, two of the most important drugs for treating pulmonary fibrosis. Among them, in AT2 cells of conditional Nedd4-2^−/−^ mice, the level of Smad2 phosphorylation induced by TGF-β stimulation decreased after pirfenidone pretreatment (Figure 3A). Furthermore, research findings demonstrate the effects of pirfenidone on downstream target genes of the TGF-β signaling pathway, including Serpine1, Smad7, and Skil. Pirfenidone pretreatment restored TGF-β–induced gene expression to levels seen in controls, indicating that it suppresses pro-fibrotic transcription by blocking the TGF-β pathway (Figure 3B–D). Ultimately, curbing fibroblast expansion and myofibroblast transition while dampening pulmonary inflammation [149]. Simultaneously, nintedanib attenuates inflammation and fibrosis by inhibiting receptor tyrosine kinases in multiple signaling pathways. Moreover, PTMs have emerged as a pivotal element in advancing combination therapies for pulmonary diseases. Studies have shown that synergistic therapy with nintedanib and programmed death ligand 1 (αPD-L1) has significant anti-pulmonary fibrosis effects. Nintedanib elevates PD-L1 expression, which, via STAT3 phosphorylation, boosts Immune Checkpoint Inhibitors (ICI) responsiveness, eases pulmonary complications, and remodels the antitumor immune milieu [150], suggesting the possibility of combined treatment for pulmonary fibrosis and tumors. Beyond that, PTM-targeted agents also curb fibrotic lung remodeling. For example, Glucagon-like peptide-1 receptor (GLP-1R) agonist liraglutide suppresses NLRP3 inflammasome activity, curbs p300-driven histone lactylation, and halts silica-exposed macrophage-conditioned medium-induced fibroblast activation [151]. Moreover, gefitinib and fostamatinib can effectively ameliorate the progression of pulmonary fibrosis by inhibiting p-EGFR-mediated fibronectin production and p-SYK-mediated pro-inflammatory cytokine synthesis in macrophages in fibroblasts [152]. Overall, phosphorylation and lactylation are notable in the application of clinical drugs, including pirfenidone and nintedanib. At the same time, they have a substantial effect on combination therapies for pulmonary fibrosis with diabetes and tumors.

However, clinical drugs at this stage can only delay the decline in lung function to a certain extent, but cannot reverse the fibrosis that has already developed. There are also some adverse reactions, such as gastrointestinal discomfort and skin rash. Therefore, pulmonary fibrosis can be improved by more comprehensively studying proteomics-targeted therapy combined with PTMs. More specifically, PTMs can alleviate pulmonary fibrosis by altering the signaling pathways that affect pulmonary fibrosis-related signals. Among them, the most prevalent strategy centers on blocking fibroblast activation via the TGF-β/Smad axis. For instance, H3K18 lactylation drives H3K18la-marked transcription to augment TGF-β1 release [119]. Pretreatment with the LDHA inhibitor GNE-140 was found to be effective in alleviating histone lactylation and thus pulmonary fibrosis [117]. PIAS4 also inhibits TGF-β signal transduction by increasing the SUMOylation of SMAD3 [122]. Glycine–Glycine–Alanine (GGA) regulates TGF-β1 by inhibiting deacetylase activity to accelerate tissue remodeling in pulmonary fibrosis [144]. Along with the TGF-β/Smad signaling pathway, numerous other targets are implicated, including the MNK-induced direct phosphorylation of eIF4E at the serine 209 (Ser209) site. This provides evidence for targeting the MNK/eIF4E/ATX/β-catenin signaling pathway by employing MNK inhibitors to alleviate fibrosis [156]. Additionally, fatty acid binding protein 5 (FABP5) knockdown downregulates the phosphorylation level of GSK-3β and increases the phosphorylation level of β-catenin, resulting in inactivation of the wnt/β-catenin pathway and abrogation of the fibrotic response [157,158,159]. Low-molecular-weight fucoidan (LMWF) with a unique structure obtained from Japanese kelp can inhibit the phosphorylation of PI3K and Akt, downregulate the PI3K/AKT signaling pathway, and inhibit the progression of EMT [160]. Moreover, the STAT3 cascade is another pivotal pathway driving pulmonary fibrosis [161,162,163,164]. Specifically speaking, phosphorylated STAT3 leads to the initiation of lung fibrosis. Cyclic RNA 406,961 (circ_406961) interacts with interleukin enhancer-binding factor 2 (ILF2) to regulate STAT3 and mitogen-activated protein kinase 8 (MAPK8, JNK) to inhibit the activation of the STAT3/JNK pathway, thereby suppressing the PM2.5-induced inflammatory response and attenuating pulmonary fibrosis [165,166]. Additionally, HDAC10 serves as a major mediator of acetylation in pulmonary fibrosis, and atomization inhalation of the LPAE-HDAC10 nanocomposite is a promising therapeutic option for pulmonary fibrosis. The ROS/NF-κB pathway was identified as an important signaling pathway for attenuating oxidative stress, inflammation, and pulmonary fibrosis in silicosis with HDAC10, which inhibits the levels of oxidative stress, IKKβ, IκBα, and p65 phosphorylation, and inflammation (Figure 3E) [153]. On the whole, to eradicate pulmonary fibrosis, several studies targeting key signaling pathways associated with PTMs, including the TGF-β/Smad, MNK/eIF4E/ATX/β-catenin, wnt/β-catenin, PI3K/AKT, STAT3/JNK, and ROS/NF-κB pathways, have made a difference in laboratory studies. However, these studies are in the early stages, and further clinical studies are needed to confirm the feasibility of these therapeutic techniques and assess their side effects.

In addition to targeting fibrosis-related signaling pathways, several other targets can alleviate pulmonary fibrosis primarily by affecting cells involved in fibrosis. As depicted in Figure 3F–I, primary Homo sapiens and mouse lung fibroblasts are subjected to qPCR and Western Blot analysis following treatment with various concentrations of XI-011. Research indicates that XI-011 markedly decreases the expression of MDM4 in primary human myofibroblasts and murine fibrosis models, while simultaneously enhancing the levels of acetylated p53. This upregulation facilitates Fas expression in pulmonary myofibroblasts and triggers their apoptosis, without substantially impacting the viability of normal lung cells [154]. Other studies have revealed that the ubiquitin-editing enzyme A20 possesses novel cell-intrinsic functions in suppressing fibroblast activation. With A20 and DREAM representing promising druggable targets for treating fibrosis [167]. In addition, some studies have determined the role of epoxyeicosatrienoic acids (EETs) in mitigating the senescence of AECs. Specifically, 1-trifluoromethoxyphenyl-3-(1-propionylpiperidin-4-yl) urea (TPPU) inhibits EET degradation, promotes Trim25-mediated ubiquitination and reduces age-related severity of pulmonary fibrosis, suggesting a novel therapeutic target for chronic lung diseases (Figure 3J) [155]. Less common PTMs also serve as potential targets for treating pulmonary fibrosis. To illustrate, the palmitoyltransferase inhibitor 2-bromopalmitate (BP, 10 μM) can antagonize pathological pulmonary responses to RSV + Cd by blocking protein S-palmitoylation (Pr-S-Pal) [168]. Moreover, histone H2A and H4 N-terminal citrullination also constitutes a tractable therapeutic node: therapeutic anti-citrullinated protein antibodies (tACPA) lower NET generation and can trigger macrophage-driven NET clearance in vivo, curbing fibrotic lung disease [169,170].

Consequently, diagnostic and therapeutic strategies targeting PTM in the management of pulmonary fibrosis integrate interventions into disease mechanisms, enhance drug specificity and efficacy, reduce systemic side effects, and support the development of innovative treatments. To elaborate further, PTMs can target fibrosis-associated cells such as fibroblasts, myofibroblasts, and epithelial cells, providing precise interventions against pulmonary fibrosis by modulating cell activation and function. Although clinical studies remain limited at this stage, these advantages make PTM-targeted therapeutic techniques promising for future clinical applications in the treatment of pulmonary fibrosis.

## 6. Conclusions and Prospects

Pulmonary fibrosis is a chronic, multifactorial disease driven by crosstalk among diverse cell types and signaling networks. Over the past few years, PTMs have emerged as pivotal regulators of pulmonary-fibrosis initiation, progression, and therapy, drawing intense investigative focus. In particular, PTMs have a strong influence on the different stages of pulmonary fibrosis by regulating various cellular functions, such as the inflammatory response, fibroblast activation, ECM deposition, and tissue repair. In this review, the mechanisms of PTMs in the injury, inflammation, and repair stages of pulmonary fibrosis were discussed in detail, and potential diagnostic markers and therapeutic targets based on PTMs were summarized. We found that PTMs’ signatures mirror the molecular pathology of pulmonary fibrosis and can serve as biomarkers for early detection and disease tracking. Furthermore, interventions that block the enzymes or reprogram the modified targets open fresh therapeutic avenues for this disease. However, the study of PTMs in pulmonary fibrosis is in its early stages, and many questions remain to be addressed. For example, the specific mechanism of action of PTMs in different cell types has not been fully elucidated, and the interactions between PTMs and their combined effects on cell function need to be further studied. Following this, large-scale, rigorously controlled trials remain essential to validate both the efficacy and safety of PTM-targeted therapies before they can enter routine clinical use. Without doubt, translation from basic research to clinical application has many challenges, including the stability of biomarkers, the standardization of assays, and the toxicity of therapeutic drugs, and these practical issues in translational medicine were not adequately discussed in this review. Therefore, future studies should focus on the following aspects. Primarily, the specific mechanism underlying the action of PTMs in pulmonary fibrosis should be comprehensively investigated. Using multiomics technology, the dynamic changes in PTMs in different cell types and tissues should be comprehensively analyzed to reveal their key nodes in the development of this disease. Thereafter, diagnostic tools and therapeutic drugs based on PTMs that can be used in the clinic need to be further developed. The combination of bioinformatics and molecular biology techniques can be used to screen and validate PTM markers and targets with clinical application value and promote the development of diagnostic reagents and drugs based on PTMs. Ultimately, the interactions between PTMs and other pathological mechanisms should be investigated. To study the relationships between PTMs and pathological mechanisms such as oxidative stress, cellular senescence, and the immune response, the similarities and differences between PTMs in pulmonary fibrosis and other fibrotic diseases need to be further studied to provide a theoretical basis for comprehensive treatment. To summarize, PTMs show great potential for application in the study of pulmonary fibrosis. Elucidating how PTMs operate and translate into clinical utility promises to deliver fresh diagnostic, therapeutic, and prognostic tools for pulmonary fibrosis—advances that could enhance patient survival and quality of life while propelling medical science forward.

## Figures and Tables

**Table 1:** Definition, formation and main function of PTMs in relation to their role in the lung.

Type	Modification	Function	References
Phosphorylation	Ser, Thr and Tyr	Energy and metabolism regulation, ECM deposition, fibroblast-to-myofibroblast transformation	[23,24,25,26,27,28,29,30]
Acetylation	Lys	Energy, autophagy and cell-cycle regulation	[31,32,33,34,35,36,37]
Methylation	Lys, Arg, His, Asp	Cell cycle, cell microenvironment, self-renewal of MPCs, fibroblast-to-myofibroblast conversion	[38,39,40,41,42,43,44,45,46,47,48]
Ubiquitination	Lys	Macromolecule stability, ferroptosis, collagen deposition, EMT	[49,52,53,54,55]
Glycosylation	Asp, Ser and Thr	Cell ferroptosis [49] and interaction	[50,56,57,58,59,60,61]
SUMOylation	Lys	Inflammatory factor release	[62,63,64,65,66,67]
Lactylation	Lys	Metabolic reprogramming	[51,68,69,70,71]
Palmitoylation	Cys	Energy and metabolism regulation, NLRP3 transfer, pyroptosis	[72,73,74,75,76,77]
Succinylation	Lys	Mitochondrial dysfunction, energy supply	[78,79,80]
Citrullination	Arg	Inflammatory response	[81,82]

**Table 2:** PTMs affect different cells in the lung and thus affect pulmonary fibrosis.

Stage	Related Cells	Effects on Cells	Post-Translational Modification Types	References
Injury	AT2 ^1,2,3,5^	AT2 to AT1 conversion, AT2 cell self-renewal	Ubiquitination	[85,86,87]
		AT2 cell activity	Ubiquitination	[88,89]
Inflammation	Macrophage ^1,5^	Phenotype transformation	Ubiquitination	[92,93,94]
		Oxidative stress	Phosphorylation	[96,97,98]
		Pyroptosis	Lactylation	[99,100,101]
	Regulatory T cells ^1,2,5^	Secretion of TGF-β	Ubiquitination, acetylation, phosphorylation and glycosylation	[102,103,104,105,106,107]
	Other immune cells ^1,2,5^	NETosis	Citrullination	[108,109]
		MoDMs Accumulation	Methylation	[110,111]
Repair	Fibroblasts ^1,2,3,4^	TGF-β axis	Phosphorylation, acetylation, lactylation, glycosylation, ubiquitination, palmitoylation and SUMOylation	[112,113,114,115,116,117,118,119,120,121,122,123,124,125]
		Fibroblast senescence	S-glutathionylation	[130,131,132]
		Interactions with AT2 cells	Lactylation	[119,135]
	Lung-resident mesenchymal stem cells ^1,2^	Transformation into myofibroblasts	SUMOylation	[136,137,138]
	Myofibroblast ^1,2,5^	The number and function of myofibroblasts	Acetylation	[139,140,141,142]
	Epithelial cells ^1,2,6^	EMT	Phosphorylation, SUMOylation and Glycosylation	[90,91,143]

^1^ Cells derived from C57BL/6 mouse tissue. ^2^ Pulmonary tissue cells from IPF patients. ^3^ Normal human bronchial epithelial BEAS-2B cell line. ^4^ Cells derived from rat tissue. ^5^ Cells derived from normal human tissue. ^6^ Cells from the human alveolar basal epithelial cell line A549.

## Data Availability

No new data were created or analyzed in this study.

## Abbreviations

The following abbreviations are used in this manuscript:

IPF	Idiopathic pulmonary fibrosis
PTM	Post-translational modifications
NETs	Neutrophil extracellular traps
ECM	Extracellular matrix
AT2	Type II alveolar epithelial cells
STAT3	Signal transducer and activator of transcription 3
4FUT	Fucosyltransferase 4
L1CAM	L1 cell adhesion molecule
IFN1	Type I Interferon
NSCLC	Non-small cell lung cancer
EMT	Epithelial–Mesenchymal Transition
AT1	Alveolar Type I Cells
TPP1	Telomere protection protein 1
Calpain 1	Cytosolic calpain 1
ITA3	Integrin α3β1
AEC2	Alveolar epithelial cells
MoDMs	Monocyte-derived macrophages
Treg	Regulatory T cells
CS	Cigarette smoke
HIF1	Hypoxia-inducible factor 1
LR-MSCs	Lung-resident mesenchymal stem cells
PAD4	Peptidylarginine deiminase 4
ACP5	Acid phosphatase 5
GGA	Ginkgolic acid, Glycine–Glycine–Alanine
Ythdf1	YTH N6-methyladenosine RNA binding protein F1
TβR1	TGF-β type I receptor
GLI	Hedgehog/glioma-associated oncogene homolog
α-SMA	Smooth muscle α-actinin
H3K27ac	Acetylation of histone H3 lysine at position 27
HDAC	Histone deacetylase
CTD-ILD	Connective-tissue-disease–associated ILD
AGEs	Advanced glycation end-products
OTUB1	Ubiquitin aldehyde-binding protein Otubain1
αPD-L1	Programmed death ligand 1
ICI	Checkpoint Inhibitors
GLP-1R	Glucagon-like peptide-1 receptor
Ser209	Serine 209
FABP5	Fatty acid binding protein 5
circ_406961	Cyclic RNA 406961
LMWF	Low-molecular-weight fucoidan
ILF2	Interleukin enhancer-binding factor 2
MAPK8	Mitogen-activated protein kinase 8
EETs	Epoxyeicosatrienoic acids
TPPU	1-trifluoromethoxyphenyl-3-(1-propionylpiperidin-4-yl) urea
tACPA	Therapeutic anti-citrullinated protein antibodies
pSmad2	Phosphorylated Smad2
MPCs	Mesenchymal progenitor cells
ME2	Mediated phosphorylation of malic enzyme 2
LAR	Lung alveolar regeneration
PPF	Progressive Pulmonary Fibrosis
METTL3	methyltransferase-like 3
lnc668	lncNONMMUT062668.2
